# EyeTribe Tracker Data Accuracy Evaluation and Its Interconnection with Hypothesis Software for Cartographic Purposes

**DOI:** 10.1155/2016/9172506

**Published:** 2016-03-21

**Authors:** Stanislav Popelka, Zdeněk Stachoň, Čeněk Šašinka, Jitka Doležalová

**Affiliations:** ^1^Palacký University, Olomouc, 77146 Olomouc, Czech Republic; ^2^Masaryk University, 61137 Brno, Czech Republic

## Abstract

The mixed research design is a progressive methodological discourse that combines the advantages of quantitative and qualitative methods. Its possibilities of application are, however, dependent on the efficiency with which the particular research techniques are used and combined. The aim of the paper is to introduce the possible combination of Hypothesis with EyeTribe tracker. The Hypothesis is intended for quantitative data acquisition and the EyeTribe is intended for qualitative (eye-tracking) data recording. In the first part of the paper, Hypothesis software is described. The Hypothesis platform provides an environment for web-based computerized experiment design and mass data collection. Then, evaluation of the accuracy of data recorded by EyeTribe tracker was performed with the use of concurrent recording together with the SMI RED 250 eye-tracker. Both qualitative and quantitative results showed that data accuracy is sufficient for cartographic research. In the third part of the paper, a system for connecting EyeTribe tracker and Hypothesis software is presented. The interconnection was performed with the help of developed web application HypOgama. The created system uses open-source software OGAMA for recording the eye-movements of participants together with quantitative data from Hypothesis. The final part of the paper describes the integrated research system combining Hypothesis and EyeTribe.

## 1. Introduction

The paper presents methodological-technical approach combining quantitative and qualitative methods which are based on specific technical tools. The aim of this paper is to introduce the newly developed technical research system and results of its validation: specifically, the creation and empirical verification of an interconnection of a web-based platform Hypothesis with an EyeTribe eye-tracking system connected to open-source software OGAMA. The interconnection was done by the creation of a new web application HypOgama.

The introduction of the paper discusses the methodology and mixed-research design (combination of quantitative and qualitative, resp., explorative methods) in the area of cognitive visualization and cartography. The paper consists of three parts which are ordered due the logic and procedure of the research system creation and verification. The first part is focused on the presentation of a tool for mass data collection: web-based platform Hypothesis. The second part of the paper presents the new low-cost eye-tracking system EyeTribe, which allows efficient realization of qualitative, respectively, explorative studies. In this part, close attention is paid to empirical study verifying the truthfulness of the low-cost EyeTribe tracker in comparison with SMI RED 250 system. The final part of the paper describes the research system which combines and integrates above-mentioned tools. Part of this last section is also an illustration of possible empirical study, where the interconnection of Hypothesis and EyeTribe for cartographic and psychology research is presented. However this case study is only an example of how the integrated research system and HypOgama application works, and it should only illustrate the procedure of conducting a mixed-research design.

A significant portion of experimental studies in the area of cognitive visualization can be sorted into two main categories. The studies in the first category monitor and record the behaviour of individuals or, rather, their conscious actions and general work methods when completing tasks with a use of a map. The most common aspects of studies are completion speed, accuracy, and correctness or frequency of a given solution (see [1–5]). The mentioned studies use a quantitative approach and subsequent statistical methods of data analysis. A second significant category is the use of eye-tracking systems. Eye-tracking studies are in many cases combined with the recording of conscious behaviour, that is, user actions (see the first category), but the crucial activities recorded are eye-movements, which offer continuous data about (even unconscious) behaviour of the participant while solving a task. In other words, the focus of the user's attention is foregrounded [6]. Due to the high processing requirements, these studies are often performed on a small sample of participants and methods other than statistical data analysis are being used, for example, explorative data analysis [7].

Eye-tracking was used for the evaluation of maps for the first time already in the late 1950s [8], but it has been increasingly used in the last ten to fifteen years. The main reasons are the declining prices of the equipment and the development of computer technology that allows faster and more efficient analysis of measured data. For usability research, eye-tracking data should be combined with additional qualitative data, since eye-movements cannot always be clearly interpreted without the participant providing context to the data [9].

An example of comprehensive research in the field of cognitive visualization by using eye-tracking is the work of Alaçam and Dalcı [10], who compared four map portals (Google Maps, Yahoo Maps, Live Search Maps, and MapQuest). The basic assumption of the study was that lower average fixation duration indicates more intuitive map portal environment. The shortest average fixation duration was found in the case of Google Maps. Fabrikant et al. [11] used eye-tracking for the evaluation of map series expressing the evolution of the phenomenon over time, or for evaluation of user cognition of weather maps [12]. Ooms et al. [13] dealt with the suitability of map label positions and differences in map reading between experts and novices. Popelka and Brychtova [14] investigated the role of 2D and 3D terrain visualization in maps.

Olson [15] compared cognitive visualization and cognitive psychology, arguing that cartographers can adapt ideas and experiments in methodology from cognitive psychologists. Equally, psychologists can use maps as stimuli in their studies. Both disciplines can examine the cognitive processes while reading and understanding maps. However, cognitive psychologists are interested in different types of cognitive processes such as attention, visual perception, memorizing, or decision-making. A map is only a tool in this context. For a cognitive cartographer, the map is far more important.

**Figure 1:**
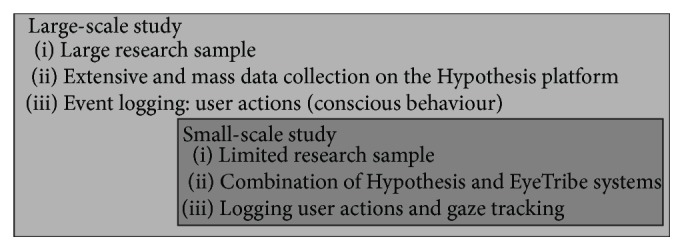
The combination of large-scale and small-scale study.

The approach mentioned above is based on close cooperation between cartographers and psychologists and shows the possibility of a connection between large-scale studies and small-scale studies based on gathering and analysing eye-tracking data. Differences between large-scale and small-scale studies are described in Figure 1.

As it is discussed in Štěrba et al. [16], using only a qualitative (explorative) or quantitative type of evaluation method is not sufficient. Therefore, it is necessary to combine those methods, enabling their suitable completion, obtaining more valid results, and achieving better interpretation. A combination of quantitative and qualitative methods was established as mixed-research design [17]. The key idea and innovation of our method are the interconnection of two approaches in the area of cognitive visualization and also finding a technological solution.

The Hypothesis platform serves primarily for the creation of experimental test batteries, online administration, and extensive data gathering. After connecting with the eye-tracking system, more detailed data on the experimental task processing methods are gathered, which allow deeper insight into the postulated cognitive processes that underlie the behavioural reactions.

Štěrba et al. [18] propose two variants of mixed-research design:

- Using the eye-tracking system for a pilot study examining a quality of experiment design with results from this pilot study being used for improvement of experiment design before large-scale data collection.
- Using Hypothesis for large-scale quantitative approach and secondary using of eye-tracking method for the subsequent specification of certain results with adjusted or changed types of tasks.

Both approaches and technical specification of Hypothesis platform are described in detail in [18] and are available online in English.

## 2. A Tool for Mass Data Collection: Web-Based Platform Hypothesis

For the purposes of large-scale experimental investigation, the creation of psychological tests, and evaluation of cartographic works, new research software concept was designed within the project “Dynamic Geovisualization in Crisis Management” [19]. Subsequently, this concept has been realized, and original software MuTeP was developed [20, 21]. MuTeP was primarily created for the purposes of objective experimental exploration and evaluation of cartographic products in the perspective of user personality.

Although MuTeP was practically proven [22], it was clear that the conception used will soon reach its limits. Another impulse for the search for a more flexible solution was an effort to involve dynamic cartographic visualization as stimuli, randomization, nonlinear test batteries, connection with eye-tracking technology, and so forth, which were not possible to implement into MuTeP software.

**Figure 2:**
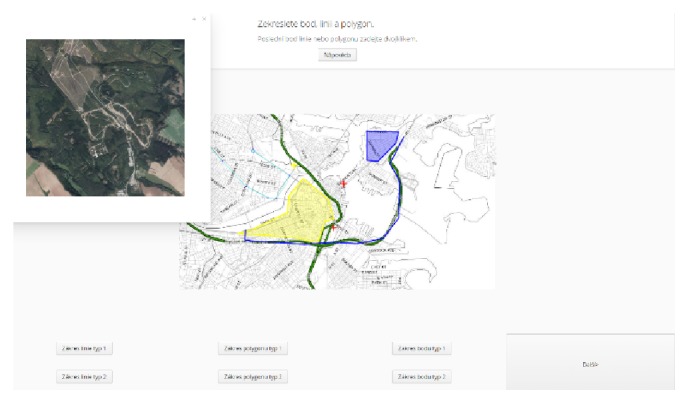
Example task on WMS interactive map. The user indicates the requested objects, draws lines, and marks out target areas by polygons. In the example shown, the user called up an orthophoto map in a dialogue-window. All the actions including the drawn point coordinates, lines, and polygons are saved in the database, and the correctness of the solution is automatically evaluated under preset conditions.

Based on experience with MuTeP and in the context of current requirements, a new software concept was designed. This new software should have the potential for long-term growth and development [23]. Hypothesis has several important advantages in comparison with MuTeP. Above all, Hypothesis enables computer adaptive testing and offers a modular solution with plugin support (such as video or interactive animation plugins) and enables the work with interactive maps (such as web map services; see Figure 2).

The technology used for designing Hypothesis consists of the following: (1) the application core and user interface are built on framework Vaadin 7; work with the database is provided by ORM Hibernate; and (2) PostgreSQL in version 9.1 (and higher) is used as a primary database system [18].

The architecture of the system is three-layer: a client, server, and database. The client part is designed for communication and interaction with the user, and its operation is provided by standard web browsers (thin client) or a special browser distributed in the application package—special Hypothesis Browser. Hypothesis Browser is based on Standard Widget Toolkit (SWT) components and ensures more strict conditions and control over running tests [18, 24].

Hypothesis works as an event-logger application, which logs all user actions and events (coordinates and timestamps of clicks, key presses, start and end time of each presented slide, exposition time of every component such as a picture or dialogue-window, zoom of maps, rotation of 3D objects, etc.). Extensive logging of user actions and events is enabled through the structure of the final slides used for the test battery (package). The package comprises the hierarchical structure of branches which contain one or more tasks, and each task contains at least one slide. The slide consists of a template and content. Such structure enables nonlinear branching of the test slides or randomization of slides. All parts of the package are stored in structured XML format. After starting a test, a selected package is loaded from the database to the server application and a new test is created. Emphasis was placed on variability and range of software usability. Figure 2 shows an example of the slide using WMS. The slide consists of two layers. The underlaying image is created with a layer: ImageLayer. Above it, there is a transparent layer: FeatureLayer, which is designed to draw demanded points, polylines, or areas by mouse and store the events [18].

**Figure 3:**
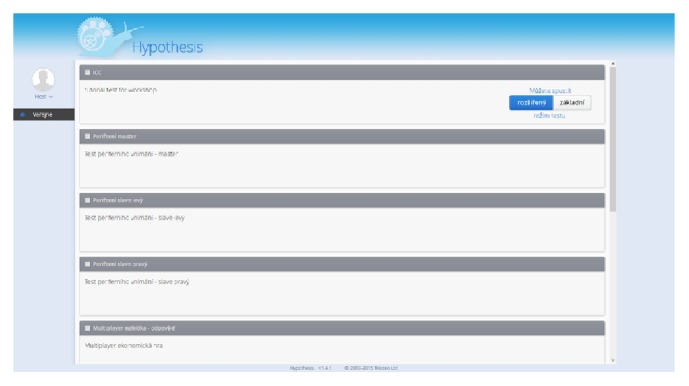
Management module in the Hypothesis platform. The user can launch the available tests in two modes: (a) legacy (launches in a normal browser) and (2) featured (launches in a controlled mode in SWT browser). The manager and the superuser have an extended access and can unlock the tests, create users, export results, and so forth.

Hypothesis is also improved with two new key functionalities that are vital for the interconnection between eye-tracking systems (or other peripherals such as EEG) and enable the realization of experiments with high reliability. These functionalities involve the use of SWT browser that allows the client to monitor and control the testing process. In other words, when using the controlled mode (see Figure 3), the participant has no way to intentionally or unintentionally exit the test by, for example, pressing alt + F4. Other common functions of web browsers are also strictly disabled, such as page refreshing or opening menus by right-clicking the mouse. The second key functionality is the recording of two time sets in the database. To avoid the problem of slow internet connection, both server time and local PC time are recorded, which means that events on the client side can be accurately synchronized (e.g., synchronizing stimulus exposition with data from the eye-tracker).

Researchers can effectively create new test batteries thanks to a combination of a number of subfunctions and tools. Emphasis is also placed on the efficiency of the software. Researchers can effectively change the content of already finished test slides and create derivatives from sample templates through the modules for user access administration and also export structured results.

Hypothesis software is freely available for collaboration on a various research topic in the Czech Republic and abroad. Access to the database and modules is provided after registration.

## 3. In-Depth Analysis of Cognitive Processes Using Eye-Tracking System

### 3.1. EyeTribe Tracker

Eye-tracking technology is becoming increasingly cheaper, both on the hardware and on the software front. Currently, the EyeTribe tracker is the most inexpensive commercial eye-tracker in the world, at a price of $99. More information about the device is available at the web page of the manufacturer (https://theeyetribe.com/). The low-cost makes it a potentially interesting resource for research, but no objective testing of its quality has been performed as of yet [25]. Dalmaijer in his study [25] with five participants compared the EyeTribe tracker with high-frequency EyeLink 1000. He states that concurrent tracking by both devices of the same eye-movements proved to be impossible, due to the mutually exclusive way in which both devices work. One of the reasons was that EyeLink uses only one eye for the recording. Delmaijer [25] also states that recording with both devices at the same time results in deterioration of results of both and often leads to a failure to calibrate at least one. Ooms et al. [26] compared EyeTribe with SMI RED 250 but also did not use the concurrent recording. In our study, we compared the EyeTribe tracker with SMI RED 250. In our case, we have not noticed any problems with calibration (see Table 1).

### 3.2. Methods of EyeTribe Accuracy Evaluation

**Figure 4:**
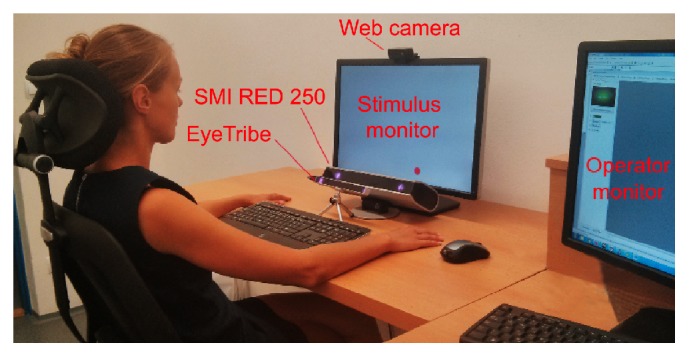
Laboratory setting for EyeTribe and SMI accuracy comparison.

For the comparison study, recording with SMI RED 250 and the EyeTribe tracker at the same time was performed. Laboratory setup is displayed in Figure 4. The EyeTribe tracker stands in front of the SMI device.

EyeTribe tracker was connected with the OGAMA software [27], where the experiment with six static image stimuli was prepared. At the same time, screen recording experiment was created in SMI experiment center (sampling frequency was set up to 60 Hz, to be the same as EyeTribe). Both devices were calibrated separately (but the eye-trackers were at their positions and turned on).

**Figure 5:**
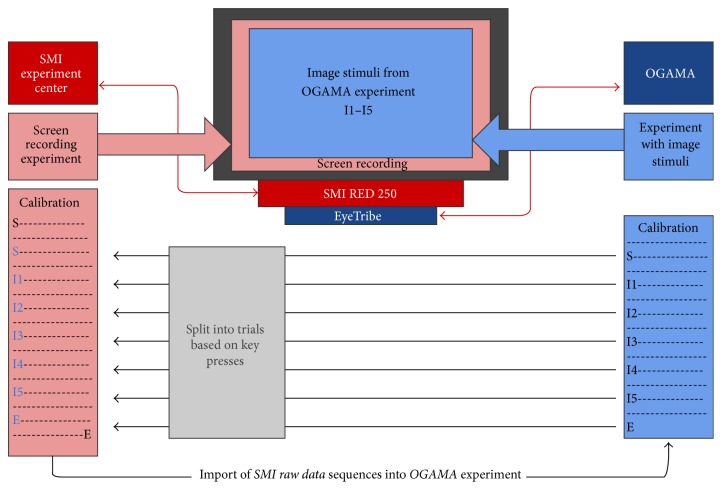
Diagram of concurrent eye-movements recording with SMI RED 250 and EyeTribe.

After calibrations, recording with SMI started. After that, experiment with static images in OGAMA was performed. That means the SMI device recorded the experiment data as well (as a screen recording video). The whole experiment procedure was done with fourteen participants. The purpose of the study was to verify how trustworthy data from EyeTribe tracker are. Recorded fixations from both eye-trackers were compared qualitatively and quantitatively. A diagram of the whole recording procedure is displayed in Figure 5.

For the comparison of recorded data from both devices, the OGAMA environment was used. Data from EyeTribe were displayed in OGAMA directly; SMI data had to be converted. For this conversion, the tool smi2ogama developed by S. Popelka was used. The tool is available at http://eyetracking.upol.cz/smi2ogama/.

The recorded screen data were cropped according to the pertinence to individual stimuli. For that, recorded key presses (for a slide change) were used.

### 3.3. Participants

**Figure 6:**
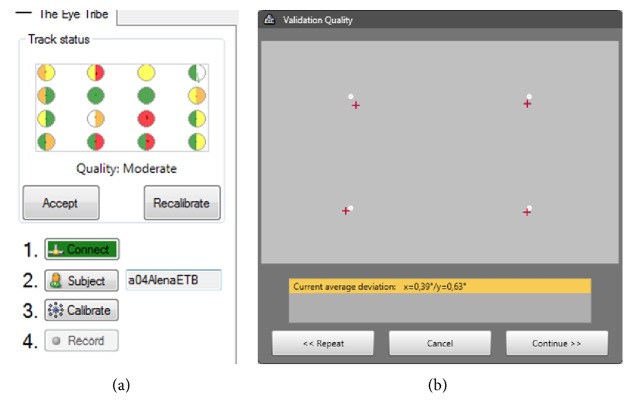
Calibration results from EyeTribe (a) and SMI RED (b) for participant “P03.”

Total of 14 respondents participated in this part of the study (ten males and four females with an average age of 29.5). They were employees and postgraduate students of department of geoinformatics. 16-point calibration was used for both devices. Results of calibration are summarized in Table 1. With the EyeTribe, it was almost not possible to achieve perfect calibration result.  Figure 6 shows the details of calibration results for participant P03. The results in OGAMA show calibration result for each of the 16 calibration points (with the use of colour); SMI shows only the average value in degrees of visual angle for axes *X* and *Y*.

For all recordings, I-DT fixation detection in OGAMA was used with the same settings. A value of 20 px was used as “maximum distance”; “minimum number of samples” was set up to 5. More information about fixation detection settings is available in [28, 29].

### 3.4. Stimuli

**Figure 7:**
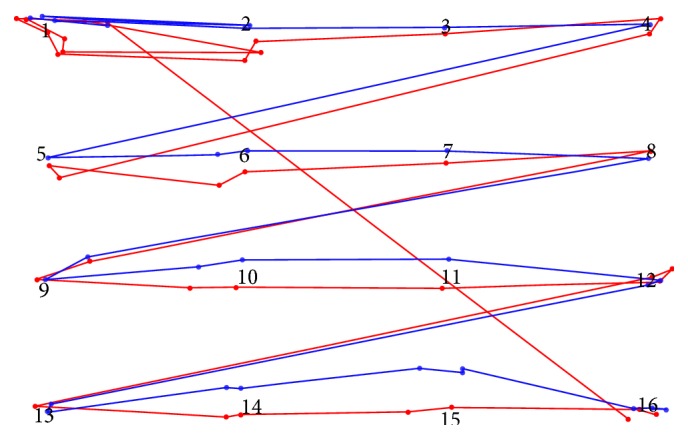
Comparison of recorded eye-movement data from participant P03 in Slide 2 from EyeTribe (blue) and SMI RED (red).

**Figure 8:**
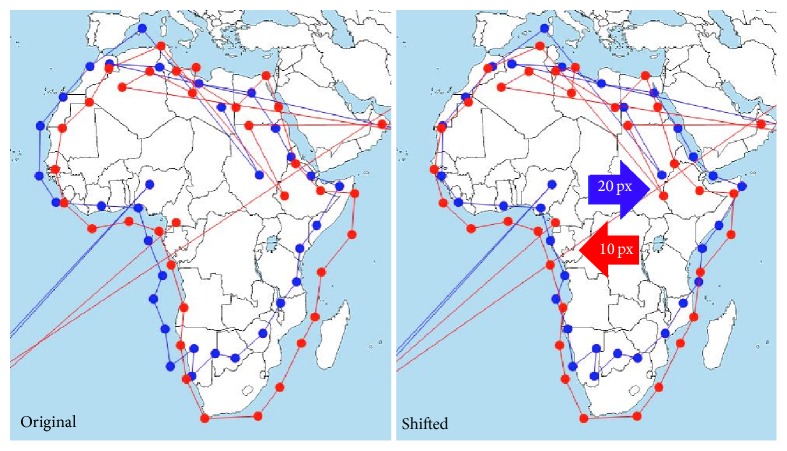
Comparison of recorded eye-movement data from participant P03 in Slide 6 from EyeTribe (blue) and SMI RED (red).

**Figure 9:**
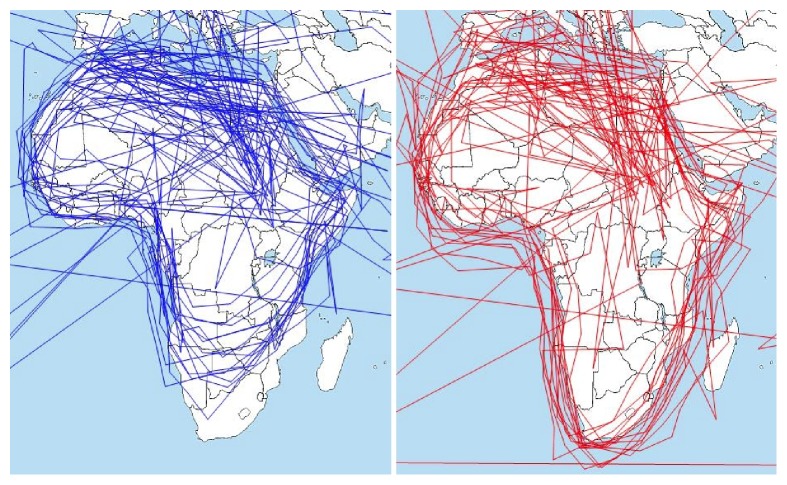
Problems with data recorded by EyeTribe (blue) at the bottom of the stimuli in comparison with SMI data (red).

The experiment contained six static images. The first one contained a grid with nine numbers; second one (Slide 2, Figure 7) contained sixteen numbers. The task of the participants was to read numbers in ascending order (from top to the bottom). Next three stimuli contained different types of maps, but the results of these stimuli are not described in this paper. The last stimulus (Slide 6, Figures 8 and 9) contained a map of the world and respondents' task was to move the eyes around Africa.

### 3.5. Results and Discussion of EyeTribe Evaluation

Eye-movement data recorded from participant P03 are displayed in Figure 7. Red points represent fixations from SMI, and blue points are fixations from EyeTribe. The task in this stimulus was only to read the numbers.

From Figure 7, it can be seen that both devices recorded around one or two fixations over each number. The accuracy of the recording is comparable. Accuracy reflects the eye-tracker's ability to measure the point of regard and is defined as the average difference between a test stimulus position and the measured gaze position [30]. The largest deviations of the EyeTribe tracker data were observed for two points in the middle of the bottom line. This situation was observed in almost all recorded data. The situation can be seen in Figure 7 in the case of points 14 and 15 (middle points in the lowest line of numbers). Gaze position recorded by EyeTribe is shifted upwards.

Another example is visible in Figure 8, which is the crop of Slide 6 stimuli. In this stimulus, the task was to move the eyes around the continent of Africa on the map. The data recorded by EyeTribe tracker were moved to the left by 20 px, but this systematic error can be corrected by a manual shift of fixations in OGAMA. This situation is depicted in Figure 8. On the left side, original data are displayed. On the right, data after horizontal shift (20 px to the right for EyeTribe and 10 px to the left for SMI) are depicted. Eye-movement data from EyeTribe for horizontally central fixations are shifted upwards, especially in the bottom part of the stimuli. See Figure 12 for more detailed analysis of fixation locations. The same issue was reported in all stimuli for most of the participants. Visualization of gaze trajectories of all participants is in Figure 9. The solution for dealing with this inaccuracy is to avoid placing important parts of the stimulus to the bottom of the screen. It will be possible to compare recorded raw data, but, in cartographic research, fixations are used for analysis, so it was more meaningful to compare fixations (identified with the same algorithm).

As an alternative for the comparison of raw data, comparison of data loss was performed. In the case of SMI recordings, average data loss (samples with coordinates 0, 0) was 0.57% of all recorded data. With the EyeTribe, the average data loss was 1.22%. Although the value is more than twice higher than in the case of SMI, it is still acceptable.

**Figure 10:**
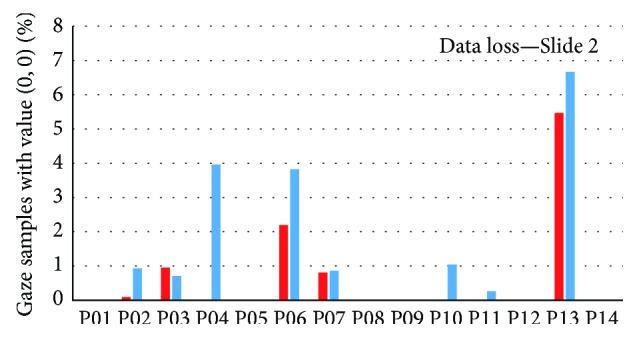
Comparison of data losses of fourteen participants during observation of Slide 2. Red bars represent SMI RED 250; blue ones represent EyeTribe tracker.

The graph in Figure 10 shows the percentage of data loss for Slide 2. It is evident that data loss is higher in the case of EyeTribe recordings, but, in most cases, less than 2% of data is missing. The highest values were observed for participants P06 and P13. Participant P06 had the worst calibration from all respondents. Participant P13 has worn glasses which can possibly cause the high data loss.

**Figure 11:**
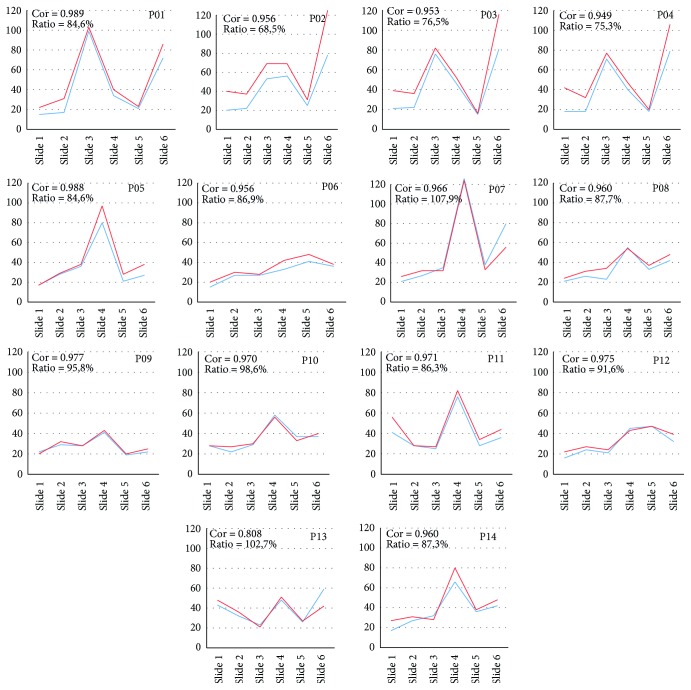
Comparison of fixation count eye-tracking metric for fourteen participants. EyeTribe data are displayed as blue line; SMI data are displayed as red line.

In the next step of accuracy evaluation, values of eye-tracking metric fixation count recorded by SMI RED 250 and the EyeTribe tracker were compared for all six stimuli in the experiment. A summary of the results is shown in Figure 11. The correlation between numbers of detected fixations was between 0.949 and 0.989 with the exception of participant P13 with the correlation of 0.808. The ratio between a number of recorded fixations with SMI device and EyeTribe was also investigated. On average, EyeTribe recorded 88.2% of fixations that were recorded by SMI device. The correlation and ratio values for each participant are presented as part of Figure 11.

**Figure 12:**
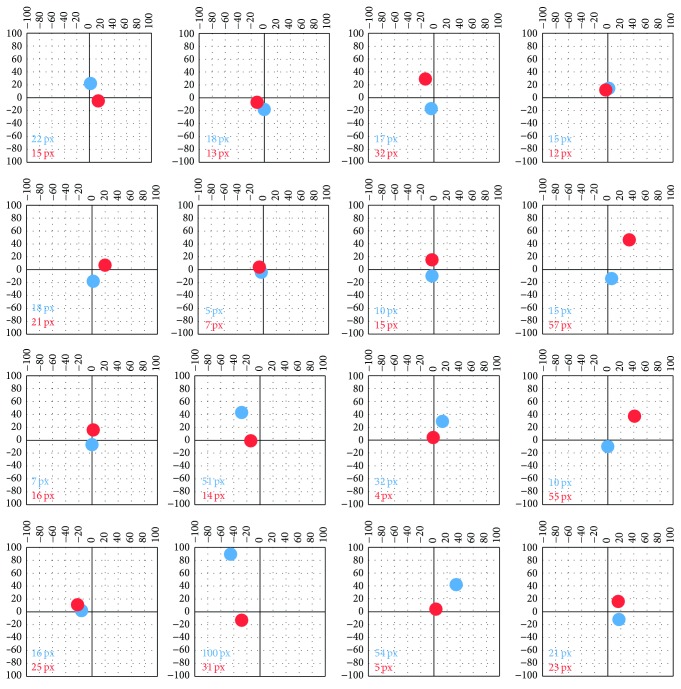
Comparison of fixation positions in Slide 2 for fourteen participants. Distance from the center of the image shows fixation deviation in pixels. EyeTribe data are displayed as blue dots; SMI data are displayed as red dots.

Beside the number of fixations, their location was compared. For this evaluation, Slide 2 with a grid of 16 numbers was chosen (Figure 7). For each participant, the deviations between coordinates of the target (number) and closest fixation were calculated. The graphs in Figure 12 show the median size and direction of the deviation for each of the 16 targets in the stimuli. It is evident that the largest deviations (heading upwards) for EyeTribe were observed for the points in the bottom part of the image (numbers 14 and 15). Each graph contains the value of the Euclidean distance of median deviations from the origin. Average deviation was 26 px for EyeTribe and 22 px for SMI.

The evaluation of truthfulness was performed on fourteen participants. According to Nielsen [31], this number should be sufficient. The evaluation of qualitative (Figures 7, 8, and 9) and quantitative (Figures 10, 11, and 12) data indicates that accuracy of low-cost EyeTribe tracker is sufficient for the use in cartographic research. Similar results were found by Ooms et al. [26], who measured the accuracy by the distance between recorded fixation locations and the actual location.

The limitation of the low-cost device is the sampling frequency, which is only 60 Hz (compare with 250 Hz of SMI RED eye-tracker). Another problem is shift of fixation locations in the bottom part of the screen. Taking into account described limits of the device, the EyeTribe may be an appropriate tool for cartographic research.

## 4. Integrated Research System: Interconnection of Hypothesis Software and EyeTribe

As one of the practical applications of the mixed-research experiment design, the Hypothesis software interconnected with the EyeTribe tracker was chosen. For the recording of eye-tracking data, the OGAMA software was used because the EyeTribe tracker is intended for developers and contains no software for data recording and analysis. OGAMA has an inbuilt slide show viewer, but the range of functionality of this viewer in comparison with SW Hypothesis is quite limited. Desktop application OGAMA principally does not allow working with web-based interactive maps and mouse clicks are recorded but not shown. Oppositely, Hypothesis visualizes clicks and allows drawing of lines and polygons. This functionality is crucial in the context of working with maps. Because of this functionality, Hypothesis connected to OGAMA via HypOgama was used.

### 4.1. Methods of Hypothesis and EyeTribe Interconnection

For the study, a simple Hypothesis experiment containing five stimuli (intro, three pairs of maps, and last slide) was used. Participants' task was to identify the differences between the maps. Coordinates of the clicks representing differences were also recorded.

OGAMA experiment was designed with only one screen recording stimulus. OGAMA in version 5.0 can record dynamic web stimuli, but it is not possible to use slides from Hypothesis as separate stimuli.

**Figure 13:**
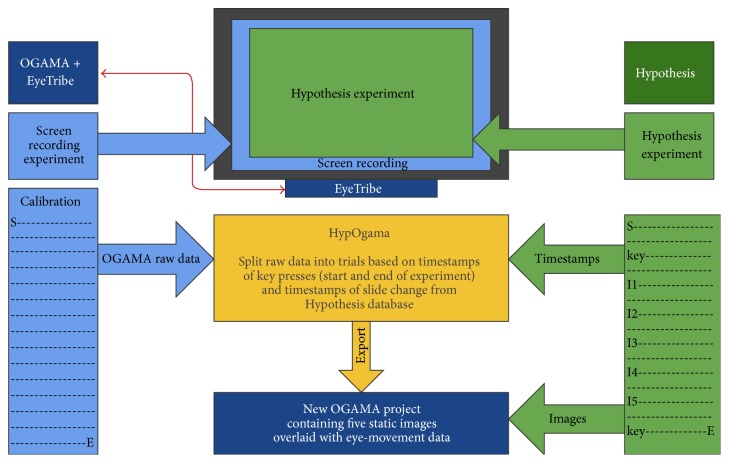
Process of splitting recorded data (screen recording) into trials with the use of HypOgama web application.

Recorded data were split according to their belonging to particular slides in the Hypothesis experiment. For the split, timestamps from Hypothesis indicating the slide change were used. The splitting and conversion of recorded data manually were time-consuming and not user-friendly. Thus, a web application called HypOgama was written in PHP for the automation of the process. The functionality of HypOgama application is illustrated in Figure 13.

**Figure 14:**
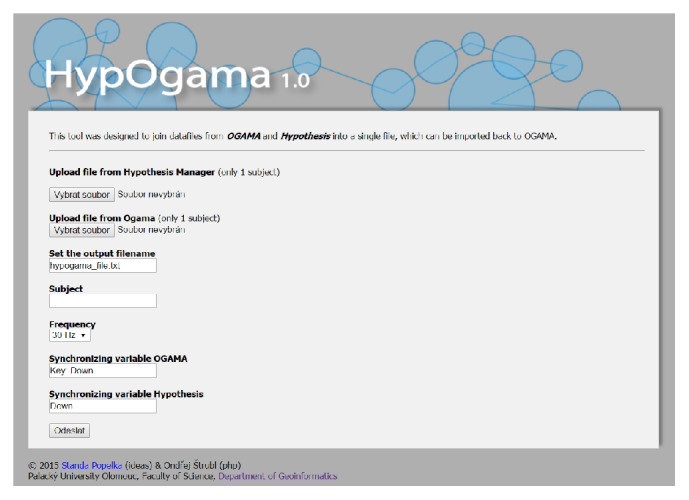
Environment of HypOgama web application.

The HypOgama application (Figure 14) is freely available at http://eyetracking.upol.cz/hypogama/.

The application synchronizes the Hypothesis time with the timestamp from the eye-tracking recording in OGAMA. The synchronization is processed by the key press that was used to start the Hypothesis experiment and which was recorded in both systems—in Hypothesis and OGAMA.

In the next step, the application scans the Hypothesis file and finds the timestamps of slide changes. These timestamps are then used for splitting raw eye-tracking data into blocks belonging to particular slides. The name of the relevant stimuli is added to all records from each block. In the final step, the data structure is modified for the direct import into a new OGAMA project.

The application contains six input fields:

1. Exported file from Hypothesis manager containing data for one participant.
2. Exported raw data from the OGAMA application for one participant.
3. Name of the output file.
4. Subject name (if blank, the ID from Hypothesis will be used).
5. Frequency of an eye-tracker (30 or 60 Hz).
6. Synchronization variables: these values indicate which key was used for the synchronization of Hypothesis and OGAMA (default value is “Key: Down” in OGAMA format and “Down” in the format of Hypothesis application).

In the Hypothesis file (ad 1), HypOgama finds the row with the key press (default Key: Down) and the corresponding time, which corresponds to the beginning of the experiment. In the next step, the column containing the slide names is scanned and the time of the first occurrence of each slide is also stored. According to this time, OGAMA recording is split. The last information obtained from the Hypothesis file is the name of the subject, overwriting the subject name in the OGAMA file.

In OGAMA file, all records prior to the synchronization key press are erased. Stimuli names are replaced by those from Hypothesis file.

Outputs of the created script are raw eye-movement data for each slide that could be directly imported into the OGAMA project. The only one necessary thing is to put image files (stimuli) into OGAMA project folder. If it is the same filename as the one contained in the Hypothesis file, images will be automatically assigned to proper data. After the whole process, a user has OGAMA project containing static image stimuli with all corresponding eye and mouse movement data. The proposed concept was applied and verified through a selected case study described below. The purpose of this short study was to illustrate the functionality of interconnection of EyeTribe and OGAMA.

For the verification of the designed process of Hypothesis and EyeTribe combination, simple test battery was designed. For chosen procedure, Hypothesis was used for large-scale quantitative approach and eye-tracking method for the subsequent specification of certain results.

The test battery was established in the Hypothesis software and was focused on verification of Gestalt principles, respectively, figure-ground organization, and on the cross-cultural comparison in the context of visual perception of cartographic stimuli [22, 32–35] on the example of specific cartographic products. The cartographic tasks were part of these more complex research batteries. The main purpose of this short cartographic study was the verification of HypOgama application and whole integrated research system for further research studies.

### 4.2. Participants

Participants of this illustrative case study were 64 students from the Masaryk University, Czech Republic, and 64 students from Wuhan University, China. In the first phase, participants were tested only on the web-based platform Hypothesis. Only a half of the dataset (Czech population) was further used in context of this particular study where the topographic and thematic maps were compared. In the second phase, the experiment was conducted with the use of eye-tracking system and the research sample is still continually extended.

### 4.3. Stimuli

The stimuli were represented by three pairs of maps that differed in 10 variables, for example, different colours of map signs, different position of the signs, and missing map signs. First two pairs of stimuli contained topographic maps. The third pair of the maps contained a thematic map.

**Figure 15:**
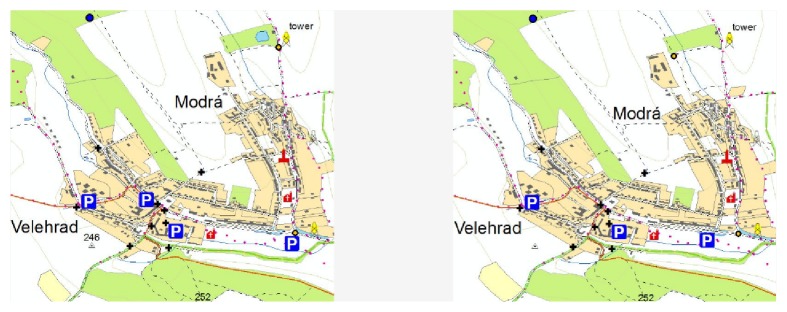
Example of stimuli—the first pair of topographic maps.

The test was structured in three main parts. In the first part, participants filled out a personal questionnaire; in the second part, a representative example of the stimuli was presented to familiarize the participants with the environment of Hypothesis. In the third part, three tasks containing pairs of stimuli described above were presented. Participants were asked to mark the differences between presented maps. The time limit for each task was 45 seconds. An example of a topographic map (Slide 1) is displayed in Figure 15. On Slide 2, similar topographic map in different scale was shown. The last slide contained thematic map (see Figure 17).

### 4.4. Results and Discussion of Hypothesis and EyeTribe Interconnection

The performed study verified stability of proposed system on long distances and, at the same time, part of the test battery was used as a pilot study to verify the functionality of an integrated research system. Stimuli comparing the effectiveness of visual search between topographic and thematic maps were selected.

**Figure 16:**
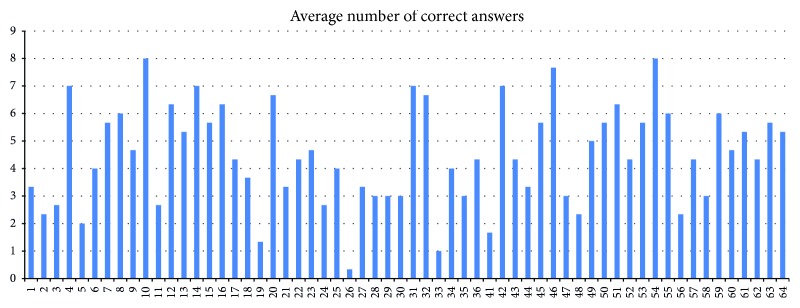
An example of results from Hypothesis. An average number of correct answers for each of the participants.

In the first phase, the test was performed in the Hypothesis application only. A number of differences identified between pairs of maps on Czech population were analysed (see Figure 16).

In the case of two pairs of topographic maps, the average number of correct answers was four. In the case of the stimuli with a thematic map, the average number of correct answers was five.

To generalize the findings, an increase of the number of maps per condition would be necessary. However, this difference was the first clue to establish working hypotheses. Based on the data from the first phase of testing, hypotheses were established only at the level of stimulus-reaction. The way of task processing by users and their solving strategies were still a black-box; thus there was a need for more detailed procedural data, especially for information about distinct search strategies.

To explore differences in the visual search, eye-tracking can be used due to the ability to provide more detailed information (e.g., which kind of object was omitted, which kind of object could be found at first glance, and which areas attracts main attention).

Therefore, in the second phase, the already used experimental battery created in Hypothesis was interconnected with OGAMA through HypOgama application and the experiment was launched with the EyeTribe system. Cartographic stimuli and the eye-tracking data were linked together and further analysed with OGAMA.

**Figure 17:**
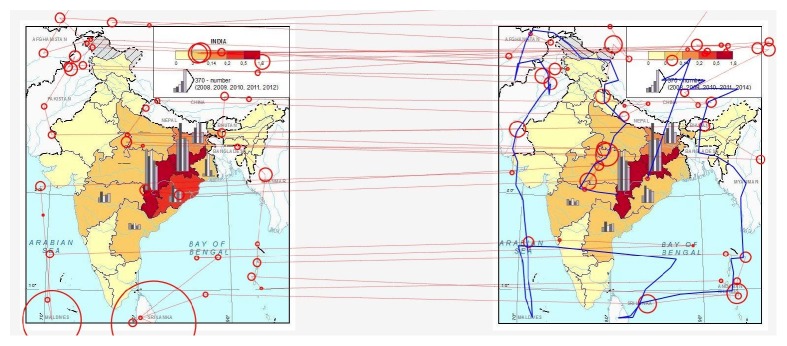
Example of eye-movement data recorded during the Hypothesis experiment. Circles represent fixations; blue line on the right is a mouse trajectory.

The example in Figure 17 shows outputs from OGAMA-scan path and mouse trajectory of one participant over the stimulus with thematic maps. In this case, fixations are distributed mainly over the text labels in the map. Participant did not find the difference in the colour of the Odisha state (on the east coast of India) under the relatively large graph. At the same time, eye-tracking metrics (e.g., fixation count, dwell time for each map, and a number of saccades between these maps) can be statistically analysed. Based on findings from both types of analyses, the hypotheses for subsequent study can be established.

The functionality of the integrated research system has been fully verified in the above-mentioned pilot study. The experiment created on the Hypothesis platform was connected with OGAMA and EyeTribe via HypOgama. Data capture including eye-tracking recording continued and exploratory analyses of these data were performed.

## 5. Conclusion

The aim of the paper was to prove the concept of the mixed-research design through the interconnection of Hypothesis (software for experiment creation, experiment execution, and data collection) and the EyeTribe tracker (the most inexpensive commercial eye-tracker). This system could prove to be a valuable tool for cognitive cartography experiments and evaluation of user behaviour during map reading process.

The first necessary step was to evaluate the accuracy of the EyeTribe tracker with the use of concurrent recording together with the SMI RED 250 eye-tracker. The results of the comparison show that the EyeTribe tracker can be a valuable resource for cartographical research.

The next part of the study was focused on the interconnection of the EyeTribe with the Hypothesis platform, developed at Masaryk University in Brno. The connection was made through a newly created web application that modifies eye-movement data recorded during screen recording experiment in the OGAMA open-source application. The application is publicly available for the community of cartographers and psychologists at web page http://eyetracking.upol.cz/hypogama.

The interconnection advantages were illustrated on an example of simple case study containing three pairs of maps. The performed case study demonstrated the ability of the combined system of the Hypothesis platform and the EyeTribe tracker to support each other and to serve as an effective tool for cognitive studies in cartography.

## Figures and Tables

**Table 1:** Summary of calibration results for all participants.

Participant	SMI *X*	SMI *Y*	EyeTribe
P01	0,4	0,2	Good
P02	0,3	0,1	Poor
P03	0,4	0,6	Moderate
P04	0,4	0,4	Perfect
P05	0,9	0,5	Good
P06	0,3	0,5	Redo
P07	0,2	0,4	Moderate
P08	0,6	0,3	Moderate
P09	0,4	0,1	Perfect
P10	0,3	0,4	Poor
P11	0,6	0,3	Poor
P12	0,5	0,5	Moderate
P13	0,3	0,3	Moderate
P14	0,4	0,6	Poor

## References

[1] Allen G. L., Miller Cowan C. R., Power H. (2006). Acquiring information from simple weather maps: influences of domain-specific knowledge and general visual-spatial abilities. *Learning and Individual Differences*.

[2] Edler D., Bestgen A.-K., Kuchinke L., Dickmann F. (2014). Grids in topographic maps reduce distortions in the recall of learned object locations. *PLoS ONE*.

[3] Kubíček P., Šašinka Č., Stachoň Z. (2014). Vybrané kognitivní aspekty vizualizace polohové nejistoty v geografických datech. *Geografie*.

[4] Nelson E. S. (1999). Using selective attention theory to design bivariate point symbols. *Cartographic Perspectives*.

[5] Nelson E. S. (2000). Designing effective bivariate symbols: the influence of perceptual grouping processes. *Cartography and Geographic Information Science*.

[6] Duc A. H., Bays P., Husain M., Kennard C., Leigh R. J. (2008). Eye movements as a probe of attention. *Progress in Brain Research*.

[7] Andrienko N., Andrienko G. (2005). *Exploratory Analysis of Spatial and Temporal Data. A Systematic Approach*.

[8] Enoch J. M. (1959). Effect of the size of a complex display upon visual search. *Journal of the Optical Society of America*.

[9] Hyrskykari A., Ovaska S., Majaranta P., Räihä K.-J., Lehtinen M. (2008). Gaze path stimulation in retrospective think-aloud. *Journal of Eye Movement Research*.

[10] Alaçam Ö., Dalcı M. (2009). A usability study of WebMaps with eye tracking tool: the effects of iconic representation of information. *Human-Computer Interaction. New Trends*.

[11] Fabrikant S. I., Rebich-Hespanha S., Andrienko N., Andrienko G., Montello D. R. (2008). Novel method to measure inference affordance in static small-multiple map displays representing dynamic processes. *Cartographic Journal*.

[12] Fabrikant S. I., Hespanha S. R., Hegarty M. (2010). Cognitively inspired and perceptually salient graphic displays for efficient spatial inference making. *Annals of the Association of American Geographers*.

[13] Ooms K., De Maeyer P., Fack V. (2014). Study of the attentive behavior of novice and expert map users using eye tracking. *Cartography and Geographic Information Science*.

[14] Popelka S., Brychtova A. (2013). Eye-tracking study on different perception of 2D and 3D terrain visualisation. *Cartographic Journal*.

[15] Olson J. M. (1979). Cognitive cartographic experimentation. *Cartographica*.

[16] Štěrba Z., Šašinka Č., Stachoň Z., Kubíček P., Tamm S. (2014). Mixed research design in cartography: a combination of qualitative and quantitative approaches. *Kartographische Nachrichten*.

[17] Creswell J. W. (2003). *Research Design: Qualitative, Quantitative, and Mixed Methods Approaches*.

[18] Štěrba Z., Šašinka Č., Stachoň Z., Morong K., Štampach R. (2015). *Selected Issues of Experimental Testing in Cartography*.

[19] Konečný M., Březinová Š., Drápela M. V. *Dynamická Geovizualizace v Krizovém Managementu*.

[20] Kubíček P., Šašinka Č., Stachoň Z. Uncertainty visualisation testing.

[21] Stachoň Z., Šašinka Č., Kubíček P., Štěrba Z. MuTeP—alternativní nástroj pro testování kartografických vizualizací a sběr dat.

[22] Stachoň Z., Šašinka Č., Štěrba Z., Zbořil J., Březinová Š., Švancara J. (2013). Influence of graphic design of cartographic symbols on perception structure. *Kartographische Nachrichten*.

[23] Morong K., Šašinka Č. Hypothesis—online software platform for objective experimental testing.

[24] Widgets S. W. T. https://www.eclipse.org/swt/.

[25] Dalmaijer E. (2014). Is the low-cost EyeTribe eye tracker any good for research?. *PeerJ PrePrints*.

[26] Ooms K., Dupont L., Lapon L., Popelka S. (2015). Accuracy and precision of fixation locations recorded with the low-cost Eye Tribe tracker in different experimental set-ups. *Journal of Eye Movement Research*.

[27] Voßkühler A., Nordmeier V., Kuchinke L., Jacobs A. M. (2008). OGAMA (Open Gaze and Mouse Analyzer): open-source software designed to analyze eye and mouse movements in slideshow study designs. *Behavior Research Methods*.

[28] Popelka S. Optimal eye fixation detection settings for cartographic purposes.

[29] Popelka S., Doležalová J. (2015). Non-photorealistic 3D visualization in city maps: an eye-tracking study. *Modern Trends in Cartography*.

[30] Holmqvist K., Nyström M., Andersson R., Dewhurst R., Jarodzka H., Van de Weijer J. (2011). *Eye Tracking: A Comprehensive Guide to Methods and Measures*.

[31] Nielsen J. (2000). *Why You Only Need to Test with 5 Users*.

[32] Čenek J. (2015). Individualism and collectivism and their cognitive correlates in cross-cultural research. *The Journal of Education, Culture and Society*.

[33] Nisbett R. E., Choi I., Peng K., Norenzayan A. (2001). Culture and systems of thought: holistic versus analytic cognition. *Psychological Review*.

[34] Rubin E., Yantis S. (2001). Figure and ground. *Visual Perception*.

[35] Wagemans J., Elder J. H., Kubovy M. (2012). A century of Gestalt psychology in visual perception: I. Perceptual grouping and figure-ground organization. *Psychological Bulletin*.

